# Investigating the diversity of intratumoral microbiota in high-grade serous ovarian cancer with varying platinum sensitivity

**DOI:** 10.3389/fcimb.2026.1652322

**Published:** 2026-06-04

**Authors:** Xianzhong Cheng, Li Xiao, Luxin Ye, Xuening Wang, Qian Zhao, Yourong Chen, Rui Zhou, Xia Xu, Jing Ni, Wenwen Guo, Xiaoxiang Chen

**Affiliations:** 1 Department of Gynecologic Oncology, The Affiliated Cancer Hospital of Nanjing Medical University, Jiangsu Cancer Hospital, Jiangsu Institute of Cancer Research, Nanjing, China; 2 Jiangsu Key Laboratory of Innovative Cancer Diagnosis & Therapeutics, Nanjing, China; 3 Department of Obstetrics and Gynecology, People’s Hospital of Yangzhong City, Yangzhong, China; 4 Department of Chemotherapy, The Affiliated Cancer Hospital of Nanjing Medical University, Jiangsu Cancer Hospital, Jiangsu Institute of Cancer Research, Nanjing, China; 5 Department of Pathology, The Second Affiliated Hospital of Nanjing Medical University, Nanjing, China

**Keywords:** 16S rRNA, intratumoral microbiome, ovarian cancer, platinum refractory, platinum-resistant recurrent, platinum-sensitive recurrent

## Abstract

**Background:**

Ovarian cancer remains the most lethal gynecological malignancy. However, no studies have investigated the differences in intratumoral microbiota among patients with varying platinum sensitivity. This study aims to explore the intratumoral microbiota of ovarian cancer in relation to different sensitivities to platinum-based chemotherapy.

**Methods:**

Tumor samples were collected from ovarian cancer patients exhibiting different platinum-sensitive statuses and subjected to microbiome (16S rRNA gene sequencing) analyses. Following DNA extraction and PCR amplification, library construction and sequencing were performed. The differences in intratumoral microbiota across various groups were analyzed both individually and collectively using a range of bioinformatics approaches.

**Results:**

A total of 22 patients with high-grade serous ovarian cancer participated in this study, including 6 from the platinum-sensitive recurrent group, 8 from the platinum-resistant recurrent group, and 8 from the platinum-refractory group. Bacterial diversity within the intratumoral microbiota, phylogenetic profiles of microbial communities, as well as functional predictions and bacterial phenotypes all exhibited significant differences among these three groups. At the phylum level, Firmicutes, Actinobacteria, and Acidobacteria were significantly more abundant in the platinum-refractory group compared to both the platinum-sensitive recurrent group and the platinum-resistant recurrent group. Additionally, genera Lactococcus and Corynebacterium showed significant enrichment in the platinum-refractory group relative to both other groups.

**Conclusions:**

This study is pioneering in identifying variations in intratumoral microbiota associated with differing sensitivities to platinum therapy in ovarian cancer patients; these findings may provide valuable insights for future mechanistic research.

## Introduction

Ovarian cancer is one of the three most prevalent gynecologic malignancies and ranks as the fifth leading cause of cancer-related mortality among women (Bray et al., 2024; Siegel et al., 2026). Among all histologic subtypes, high-grade serous ovarian carcinoma (HGSOC) is the most common, accounting for approximately 80% of ovarian cancer–related deaths (Kim et al., 2018; Xu et al., 2022). The current standard first-line treatment consists of cytoreductive surgery followed by platinum-based chemotherapy (Chandra et al., 2019). Although ~80% of newly diagnosed patients initially respond to platinum-based regimens, up to 70% ultimately experience disease recurrence and develop acquired platinum resistance—a major contributor to poor long-term survival (Liu et al., 2022). The emergence of platinum resistance significantly limits the clinical utility of platinum-based therapy. Consequently, preventing, delaying, or reversing platinum resistance remains a central therapeutic objective in ovarian cancer management. Over the past several decades, extensive research has been devoted to elucidating the molecular underpinnings of platinum resistance in ovarian cancer; however, the mechanisms remain incompletely characterized (Yang et al., 2022). Moreover, clinically validated biomarkers capable of reliably predicting baseline platinum sensitivity—or its dynamic evolution during treatment—remain lacking.

In recent years, growing attention has been directed toward the correlation between intratumoral microbiota and clinical prognosis in solid tumors, including ovarian cancer (Liu et al., 2025; Mai et al., 2025; Yang et al., 2025; Yu et al., 2025; Zhang et al., 2025). Traditionally, the female upper reproductive tract—including the ovaries and fallopian tubes—was considered a sterile environment; however, emerging evidence demonstrates the presence of a resident microbiota in these anatomical sites (Wang et al., 2020; Qin et al., 2022). Notably, prior studies have identified compositional and functional differences between intratumoral and adjacent noncancerous ovarian tissues (Wang et al., 2020). Despite this progress, investigations into the association between the intratumoral microbiota of ovarian cancer and its biological behavior—including tumor progression, metastasis, and chemotherapy resistance—remain scarce.

Therefore, elucidating the relationship between intratumoral microbiota and platinum resistance in ovarian cancer represents both a significant unmet need and a novel research direction. To systematically investigate potential links between platinum sensitivity and the intratumoral microbiome, we conducted an integrated multi-omics study using tumor samples from patients with high-grade serous ovarian carcinoma (HGSOC). This study combined 16S rRNA gene sequencing-based microbiome profiling with untargeted metabolomic analysis, supported by comprehensive bioinformatic and statistical approaches. Furthermore, we aimed to identify key bacterial taxa most strongly associated with platinum resistance and to uncover underlying molecular and metabolic mechanisms contributing to treatment failure in HGSOC.

## Methods

### Study patients

This study was approved by the Ethics Committee of the Affiliated Cancer Hospital of Nanjing Medical University. All enrolled patients underwent surgery at the Affiliated Cancer Hospital of Nanjing Medical University and provided written informed consent. All patients included in the study were pathologically diagnosed with high-grade serous carcinoma following primary debulking surgery and subsequent platinum-based chemotherapy. Detailed baseline clinical information—including medical history, demographic and clinical characteristics, and follow-up data—was retrieved from the hospital’s electronic medical record system.

### Tissue sample obtaining and platinum sensitivity assessment

According to the follow-up data, samples from 22 patients with recurrent HGSOC were obtained at our cancer center. Samples were all collected during the preoperative period and placed in sterile tubes, stored in a -80 °C refrigerator in advance for subsequent use. The platinum-free interval (PFI)—defined as the time between the completion of the last platinum-based chemotherapy and disease recurrence—was used to stratify enrolled patients. All included patients were categorized into three groups: the platinum-sensitive recurrence (PSR) group (PFI ≥ 6 months), the platinum-resistant recurrence (PRR) group (PFI < 6 months), and the platinum-refractory (PR) group (disease progression during first-line platinum-based treatment).

### DNA extractions and PCR amplification

DNA was extracted from ovarian cancer tissues with different platinum sensitivity using cetyltrimethylammonium bromide (CTAB). Primers 27F:5’-AGRGTTTGATYNTGGCTCAG-3’ and 1492R:5’- TASGGHTACCTTGTTASGACTT-3’ were used to amplify the full-length 16S rRNA gene with specific barcodes for each sample. The PCR conditions consisted of an initial denaturation at 95°C for 2 minutes; 25 cycles of denaturation at 95 °C for 30 seconds, annealing at 55°C for 30 seconds, and extension at 72 °C for 1 minute; and then final extension at 72 °C for 5 minutes. PCR amplification was performed in a total volume of 20 μL reaction mixture containing 4 μL of 5 × FastPfu Buffer, 2 μL of 2.5 mM dNTPs, 0.8 μL of each primer (5 μM), 0.4 μL of FastPfu Polymerase, and 10 ng of template DNA, and PCR-grade water to adjust the volume (Zhao et al., 2023).

### Library construction and sequencing

In this study, 2% agarose gel electrophoresis was used to confirm the PCR products, which were purified using the AxyPrep DNA Gel Extraction Kit (Axygen Biosciences, USA). QuantiFluorTM-ST (Promega, USA) was used for quantification, and amplicon pools were prepared for library construction. Pacific Biosciences SMRTbellTM Template Prep kit 1.0 (PacBio, USA) was used to prepare SMRTbell libraries and sequenced on the PacBio RS II (LC-Bio Technology Co., Ltd., Hangzhou, China) (Zhao et al., 2023).

### Data analysis

SMRT Link (v6.0) was used to generate circular consensus sequence (CCS) reads from raw subreads with the following parameters: minPasses = 5; minPredictedAccuracy = 0.9. Then lima (v1.7.1) was used to distinguish CCS reads from different samples, and cutadapt (v1.9) was used to identify primers. CCS reads between 1200 bp and 1650 bp were retained after length filtering. After deduplication and filtering of chimeric sequences using DADA2, feature tables and feature sequences were obtained. Alpha diversity (Chao1, Observed species, Goods coverage, Shannon, Simpson) and Beta diversity were calculated by random normalization to the same sequence. ASVs were annotated by feature sequences aligned with the SILVA database (version 138). Other related analysis plots involved in the study were implemented using R packages (Zhao et al., 2023).

### Handling of environmental contaminants

All pre-PCR procedures (tissue dissection, sample handling, and DNA extraction) were performed in a dedicated Class II biosafety cabinet, which was routinely UV-sterilized and cleaned with DNase/RNase removal solution before each use. The biosafety cabinet was located in a room physically separated from post-PCR amplification areas to prevent cross-contamination. Surgical instruments (scalpels and forceps) were autoclaved and treated with DNA-OFF solution between samples. Filtered pipette tips were used exclusively, and all reagents were DNase-free and aliquoted to avoid repeated freeze-thaw cycles. Technicians wore dedicated laboratory coats, face masks, and powder-free gloves, with gloves changed frequently during sample processing to minimize human-derived microbial contamination. These measures were consistently applied throughout the entire experimental workflow.

### Bioinformatics processing and decontamination

To computationally address contaminants, we employed a two-step approach. First, we applied the decontam R package using the prevalence method (p < 0.05) to filter out amplicon sequence variants (ASVs) that were significantly more frequent in the negative controls than in the tissue samples. Second, we curated the remaining ASVs to remove sequences identified as common laboratory contaminants (e.g., Ralstonia, Cutibacterium).

## Results

### Summary of clinical characteristics

**Figure 1:**
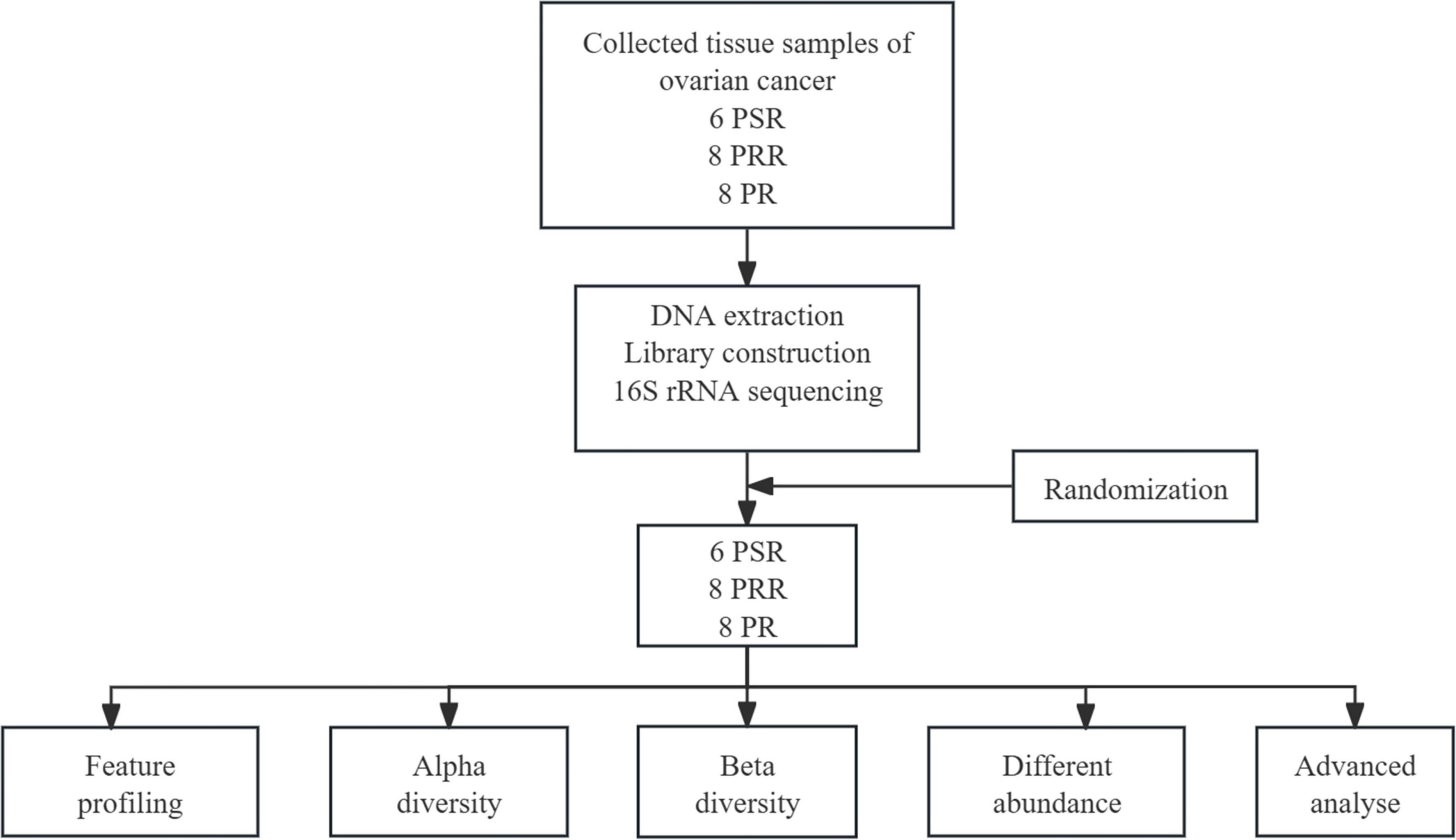
The flowchart of the research process in this study.

In total, 22 eligible patients with recurrent HGSOC were included in this study and divided into the PSR group (6 cases), PRR group (8 cases) and PR group (8 cases) according to the duration of PFI. Among the included patients, the median age was 54.5 years, the primary site was the ovary in all cases, and the histological type was high-grade serous carcinoma in all cases. Among them, 13.6% were stage I-II, and 81.8% were stage III-IV. The proportions of patients with ECOG scores of 0 and 1 were 45.5% and 54.5% respectively. Baseline characteristics was presented in **Table 1**. The flowchart shows the research process of this study (**Figure 1**).

**Table 1:** Baseline characteristics of enrolled patients. .

Characteristic	Number of patients (percent,%)
Age(years, range)	
≤54	11 (50)
>54	11 (50)
ECOG	
0	10 (45.5)
1	12 (54.5)
2	0 (0)
Primary tumor location	
Ovary	22 (100)
Fallopian tube	0 (0)
Peritoneum	0 (0)
Histological type	
High-grade serous carcinoma	22 (100)
Other	0 (0)
FIGO stage	
I-II	3 (13.6)
III-IV	18 (81.8)
Unknown	1 (4.5)
Platinum status	
Platinum sensitive	6 (27.3)
Platinum resistant	8 (36.4)
Platinum refractory	8 (36.4)

ECOG, Eastern Cooperative Oncology Group; FIGO, International Federation of Gynecology and Obstetrics.

### Bacterial diversity of the intratumoral microbiota

**Figure 2:**
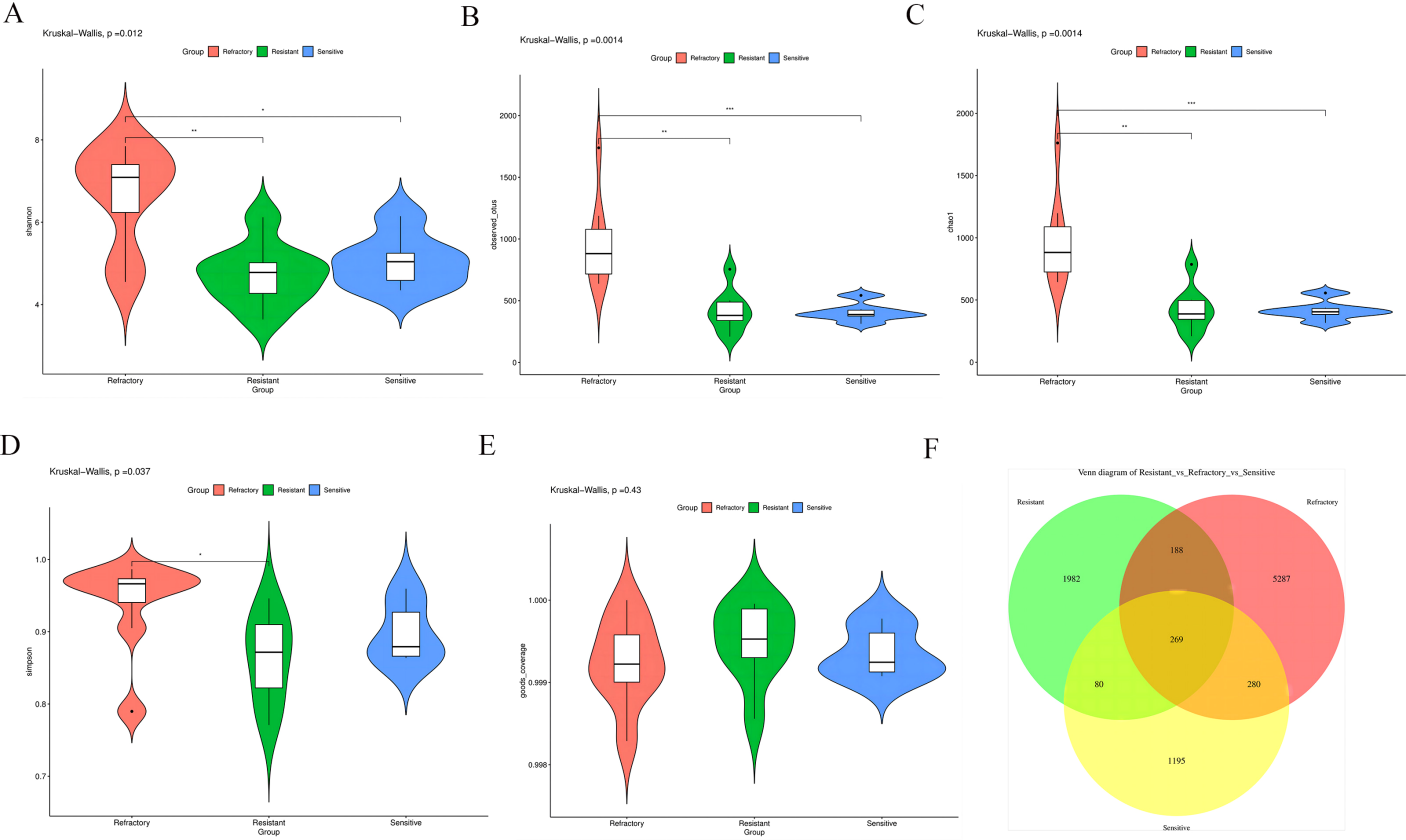
There were statistically significant differences in the Shannon (p = 0.0012) **(A)**, observed species (p = 0.0014) **(B)**, Chao1 (p = 0.0014) **(C)** and Simpson (p = 0.037) indexes **(D)**, whereas the Good’s coverage index (p = 0.43) was not significantly different between three groups **(E)**. The Venn diagram of three groups was showed in **(F)**.

The intratumoral microbiota was assessed using 16S rRNA MiSeq sequencing. A total of 1,729,922 high-quality 16S rRNA gene sequences were identified, with a median read count of 83,646 (ranging from 51,877 to 100,976) per sample. After the taxonomic assignment, 9281 OTUs were obtained. The species accumulation curve and the rarefaction curve of all samples supported the adequacy of the sampling efforts. Likewise, relative bacterial evenness was evaluated by rank abundance curves, exhibiting similar patterns in all samples (**Supplementary Figure 1**). Alpha diversity indexes were calculated to assess the differences in bacterial diversity among the three groups. To evaluate the differences in bacterial diversity between the three groups, sequences were aligned to estimate alpha diversity and beta diversity. There were statistically significant differences in the Shannon (p = 0.0012) (**Figure 2A**), observed species (p = 0.0014) (**Figure 2B**), Chao1 (p = 0.0014) (**Figure 2C**) and Simpson (p = 0.037) indexes (**Figure 2D**), whereas the Good’s coverage index (p = 0.43) was not significantly different between three groups (**Figure 2E**). The results showed that intratumoral microbial alpha diversity was significantly higher in the PR group than in the PRR and PSR groups.

Moreover, the Venn diagram showed that 269 of the total 9281 OTUs were shared among the three groups, whereas 457 of 9281 OTUs were shared between PR and PRR groups, 549 OTUs were shared between PR and PSR groups, and 349 OTUs were shared between PRR and PSR groups. Notably, 1982 OTUs were unique for the PRR group, and 1195 OTUs were unique for the PSR group, whereas 5287 OTUs were unique for the PR group (**Figure 2F**).

**Figure 3:**
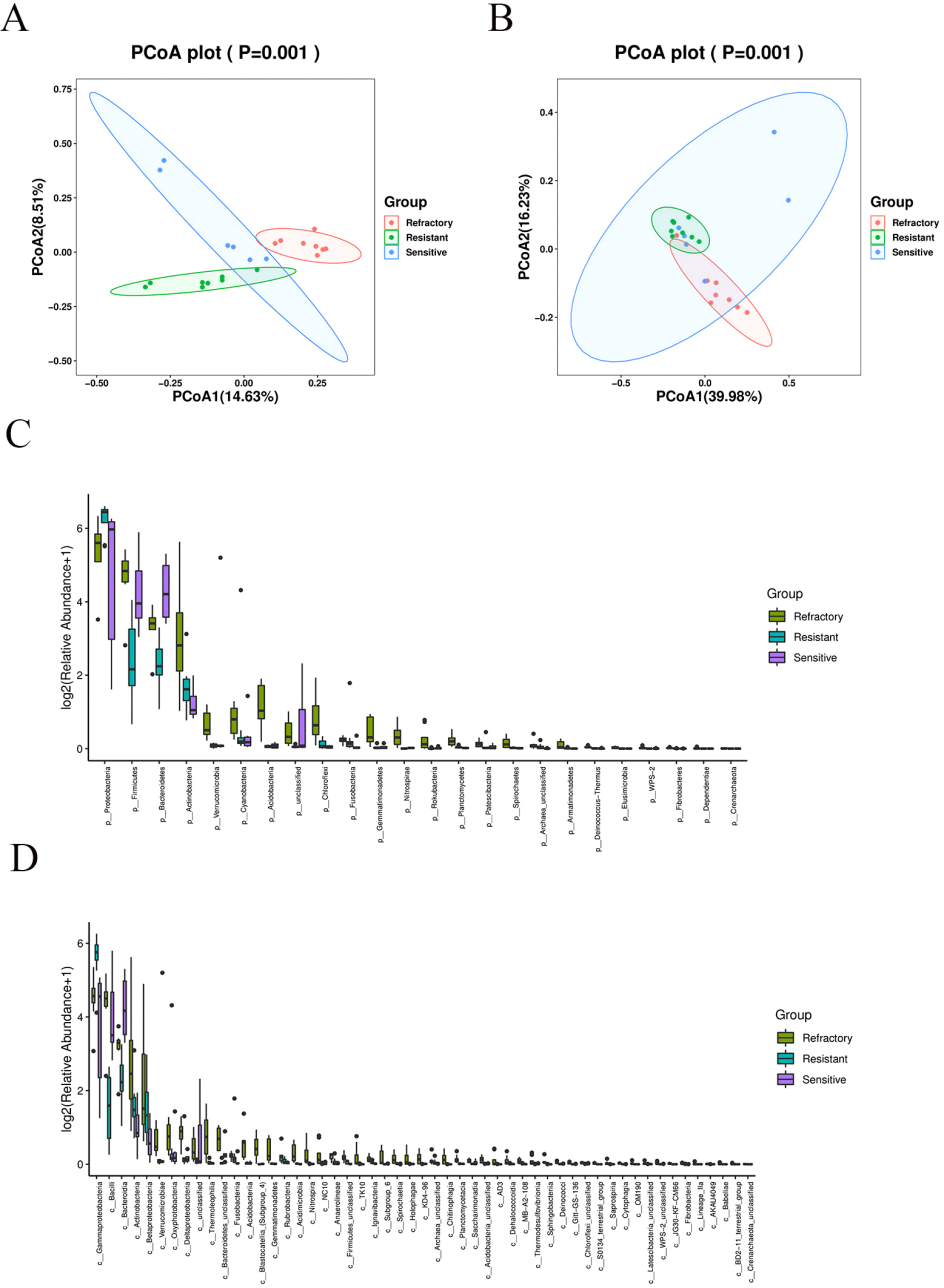
The unweighted **(A)** and the weighted **(B)** PCoA plots were performed to calculate the beta diversity. The differences at the phylum and class level are shown in **(C, D)**.

To display the microbiome space between samples, both the unweighted and the weighted PCoA plots were performed to calculate the beta diversity. The results showed a gradually separated distribution of the intratumoral microbial communities among these three groups (P = 0.001) (**Figures 3A, B**). It can be found that the abundance of Actinobacteria was the highest in the PR group, followed by the PRR group, and the lowest in the PSR group, with significant statistical differences. Furthermore, the relative abundance of p_Firmicutes was the higher in PR group. Analysis at the class, order, family, genus, and species levels was also performed (**Supplementary Figures 2A–E**), and the differences at the phylum and class level are shown in **Figures 3C, D**.

### Phylogenetic profiles of intratumoral microbial communities

**Figure 4:**
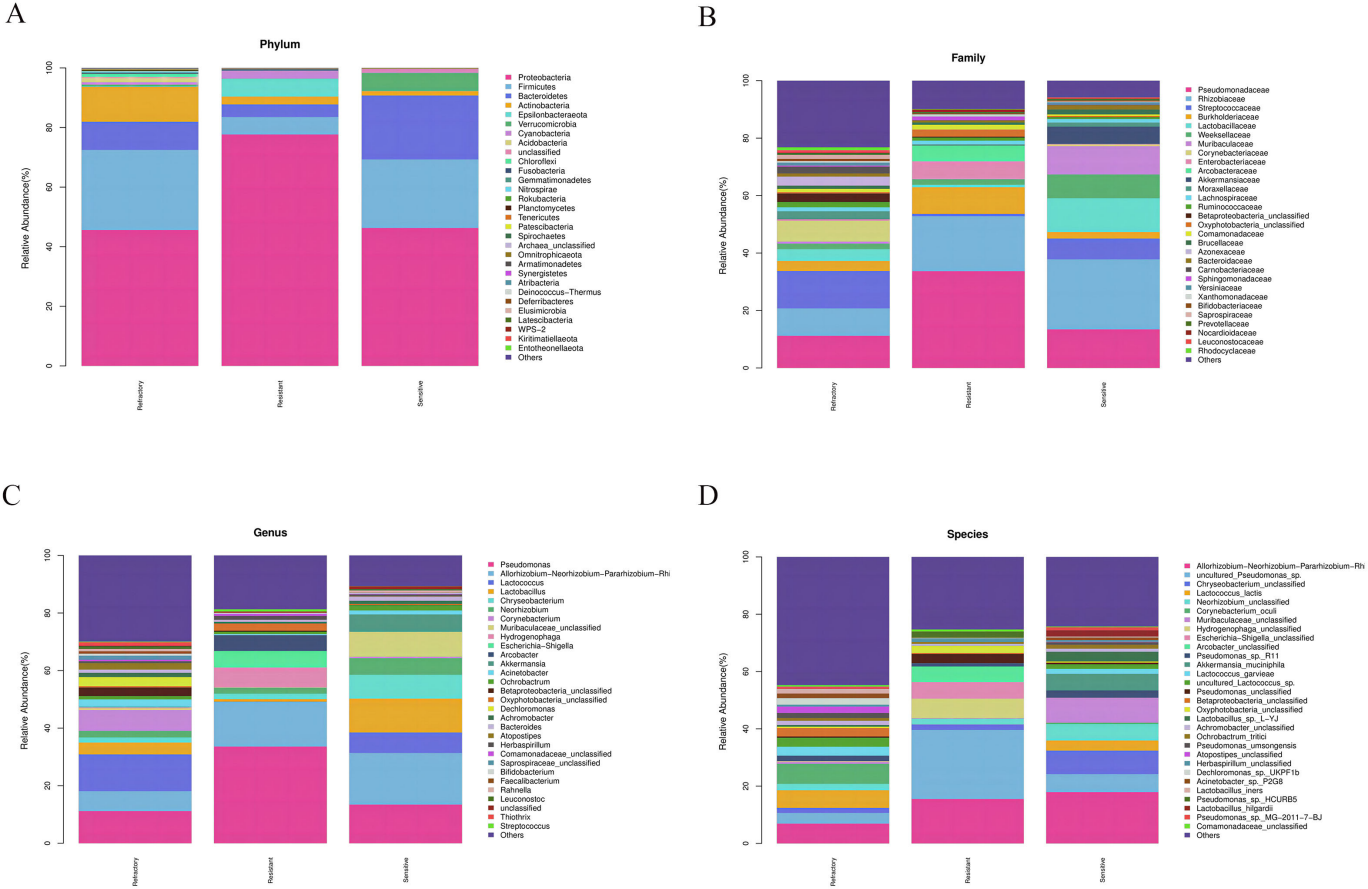
The average composition of bacterial communities at the phylum **(A)**, family **(B)**, genus **(C)**, and species **(D)** levels.

The average composition of bacterial communities at the phylum, family, genus, and species levels was shown in (**Figures 4A–D**), respectively. Proteobacteria, Firmicutes, Bacteroidetes, Actinobacteria, Epsilonbacteraeota and Verrucomicrobia were the six dominant bacterial phyla in the three groups. At the phylum level, Firmicutes, Actinobacteria and Acidobacteria were significantly higher in the PR group than in PSR and PRR groups. Bacteroidetes and Verrucomicrobia were significantly higher in PSR group than other two groups, while Proteobacteria, Epsilonbacteraeota and Cyanobacteria were higher in PRR group. Bacterial abundance at the genus level was also compared between three groups. Genus Lactococcus and Corynebacterium were significantly enriched in PR group compared with PSR and PRR groups. Genus Pseudomonas, Hydrogenophaga, Escherichia-Shigella and Arcobacter were significantly higher in PRR group, while Allorhizobium-Neorhizobium-Pararhizobium-Rhizobium, Lactobacillus, Chryseobacterium, Neorhizobium, Muribaculaceae_unclassified and Akkermansia were higher in PSR group (**Supplementary Figure 3**).

**Figure 5:**
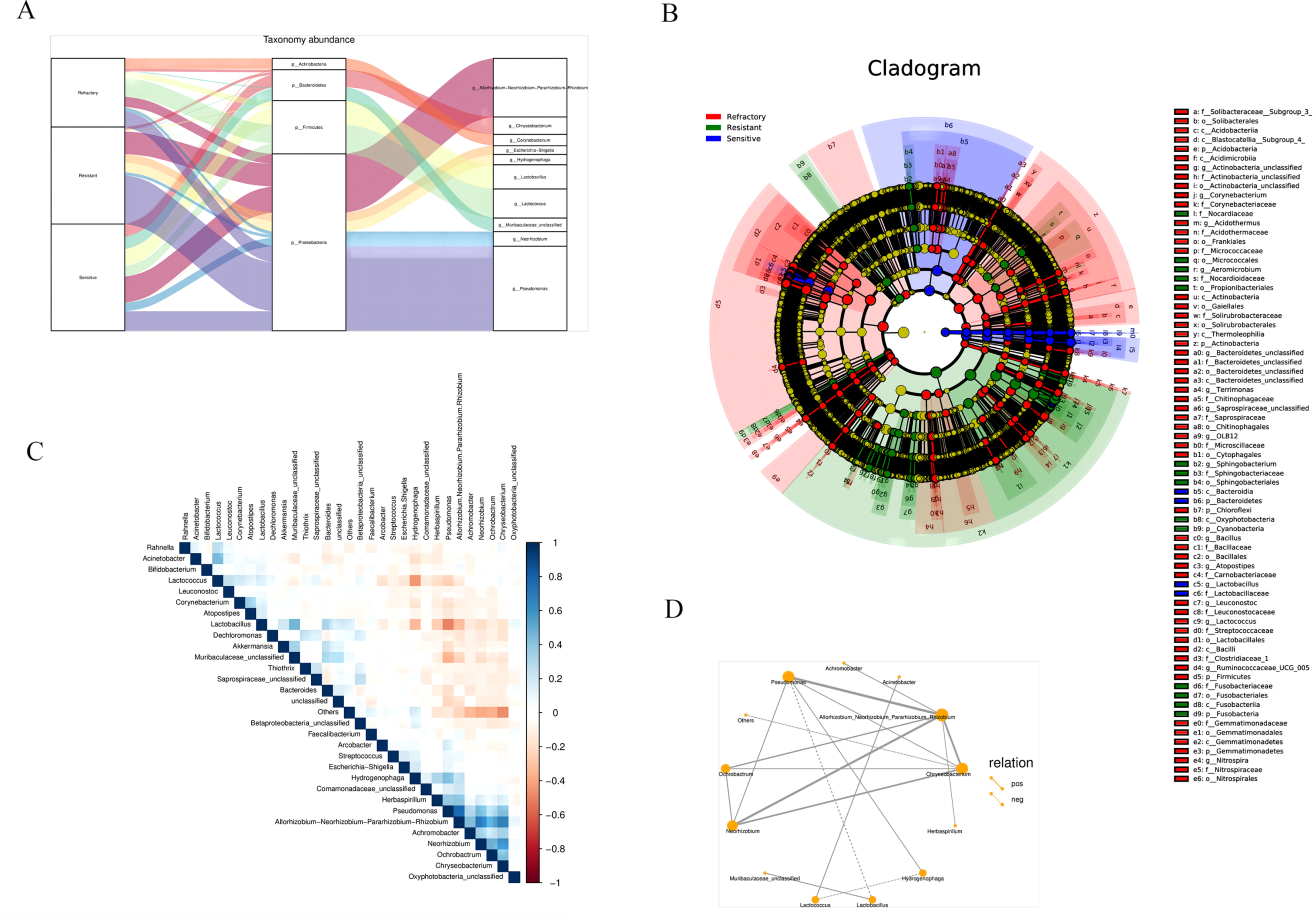
Sankey analysis based on the phylum and species levels of intratumoral microbiota **(A)**; the results of Linear discriminant analysis Effect Size (LefSe) **(B)**; a heat map used to analyze the correlation between 31 genera and platinum resistance **(C)**; network analysis based on 13 genera **(D)**.

Sankey analysis based on the phylum and species levels of intratumoral microbiota showed that Actinobacteria were specifically expressed in PR ovarian cancer tumors, and the corresponding species level was Corynebacterium. The results suggested that Corynebacterium Bacilli are almost always present in platinum-refractory ovarian cancer tumors. In addition, p_Firmicutes and g_Lactococcus was also more abundant in PR group, as shown in **Figure 5A**.

The results of Linear discriminant analysis Effect Size (LefSe) suggest that p_Actinobacteria, p_Firmicutes, c_Actinobacteria, c_Bacill, g_Lactococcus, g_Corynebacterium, s_Corynebacterium_oculi and s_Lactococcus_lactis are all platinum-refractory ovarian cancer-specific bacteria, with the most obvious differences. Based on LefSe analysis, we also found that PR ovarian cancer had a richer intratumoral microbiota, followed by the PRR group PSR group (**Figure 5B**, **Supplementary Figure 4**).

**Figure 6:**
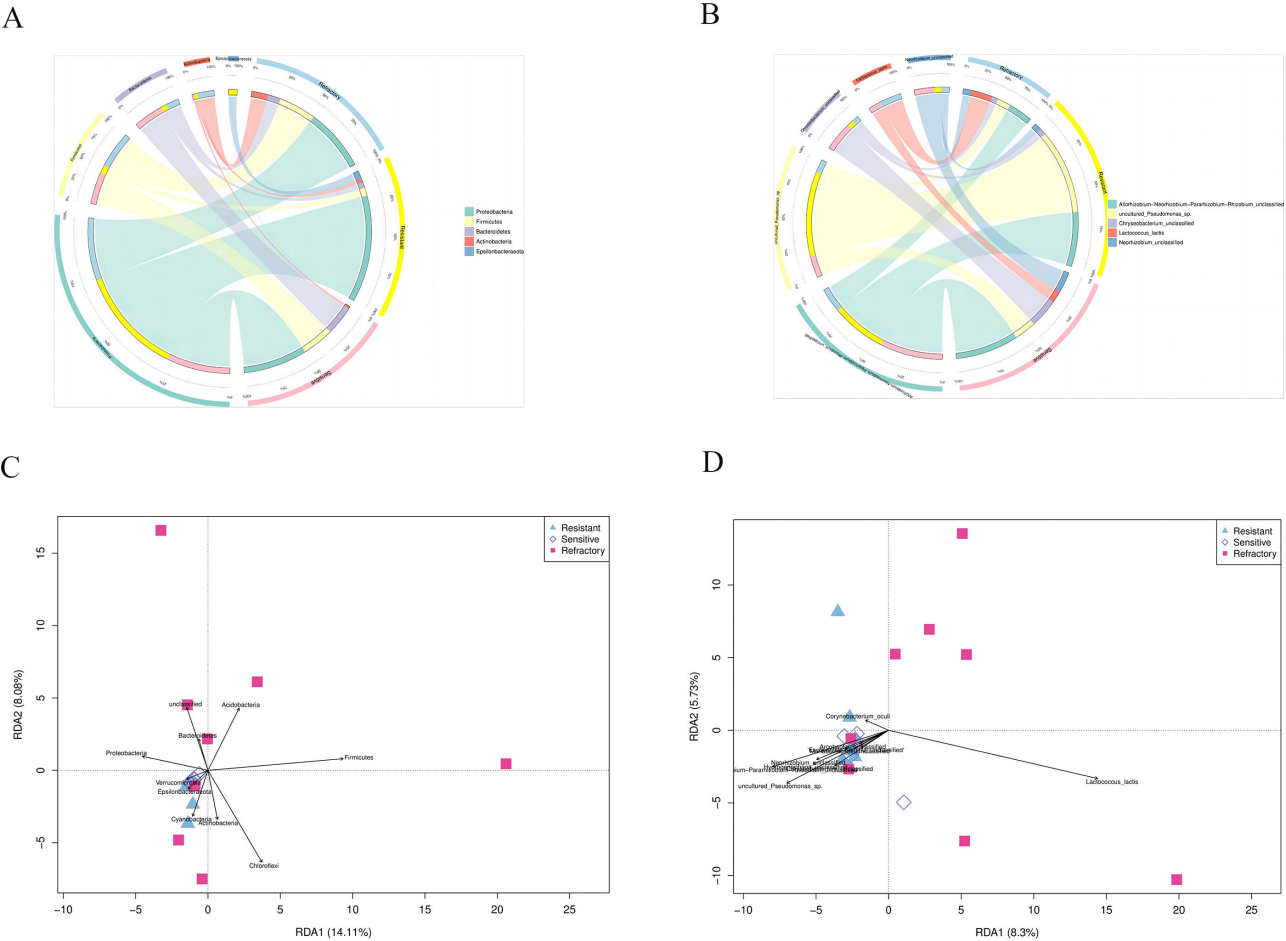
Circus plot were also performed, as shown in **(A)** (phylum level) and **(B)** (species levels). In addition, the RDA result also showed the relationship between concrete bacteria and platinum resistance **(C, D)**.

In order to characterize the correlation between bacteria at different genus levels and platinum resistance in ovarian cancer, a heat map was used to analyze the correlation between 31 genera and platinum resistance. The results showed that Acinetobacteria, Bifidobacterium, Lactococcus, Leuconostoc and Corynebacterium at genus level are all significantly related to platinum resistance (**Figure 5C**). To further explore the interactive effects of different genera, network analysis based on 13 genera showed that there were interactions between some bacteria, as shown in **Figure 5D**. To further confirm these findings in HGSOC patients, circus plot were also performed, as shown in **Figure 6A** (phylum level) and **Figure 6B** (species levels), which were similar with results showed above. In addition, the RDA result also showed the relationship between concrete bacteria and platinum resistance. The RDA result suggested that Firmicutes at the phylum level and Lactococcus lactis at the species level are the most important bacteria promoting platinum resistance, especially in PR patients (**Figures 6C, D**).

### Analysis of differential expression of intratumoral microbiota from the perspective of functional prediction and bacterial phenotype

Microbiota imbalance induces systematic metabolic alterations, while metabolic dysfunction can in turn influence microbiota composition. To study the functional and metabolic changes in microbial communities, all OTUs were aligned into the Phylogenetic Investigation of Communities by Reconstruction of Unobserved States (PICRUST) built-in reference database. PICRUST analysis identified 30 GOG pathways, 30 EC pathways (**Supplementary Figure 5**), 30 KO pathways (**Supplementary Figure 6**) and 30 TIGRFAM pathways (**Supplementary Figure 7**) with significant differential abundance between these three cohorts.

Interestingly, the KEGG homologous EC level results showed that the metabolic enzyme quinone oxidoreductase (NAD (P)H: quinone oxidoreductase, NQO) was highly expressed in PR and PRR groups, as shown in the **Supplementary Figures 5A, B**. Phenotypic analysis based on BugBase were also conducted, the phenotypes of which are divided into nine types: aerobic, anaerobic, mobile element-containing, facultative anaerobic, biofilm formation, Gram-negative, Gram-positive, potential pathogenic and stress tolerance (**Supplementary Figure 8**). BugBase results suggested that the abundance of Gram-positive and facultative anaerobic bacteria is highest in the PR group, followed by the PRR group, and lowest in the PSR group. This further suggested that Gram-positive and facultative anaerobic bacteria are closely related to platinum resistance in HGSOC. As reported above, Lactococcus lactis at the species level, one Gram-positive and facultative anaerobic bacterium, was closely associated with platinum resistance in HGSOC.

## Discussion

Ovarian cancer remains one of the most lethal gynecologic malignancies, with prognosis primarily dictated by the extent of cytoreductive surgery and the tumor’s platinum sensitivity (Hotton et al., 2025; Wang et al., 2025). Consequently, elucidating the mechanisms underlying platinum resistance—and ultimately overcoming this resistance—represents a critical therapeutic objective in ovarian cancer management. Platinum resistance is driven by a highly complex and multifactorial network, encompassing multidrug resistance (MDR), dysregulation of the DNA damage response and repair (DDR) pathways, metabolic reprogramming, oxidative stress, aberrant cell cycle regulation, enrichment of cancer stem cells (CSCs), immune evasion, impaired apoptotic signaling, dysregulated autophagy, and activation of abnormal oncogenic signaling cascades (Apelian et al., 2025; Li et al., 2025; Sideris et al., 2025).

Recent studies have confirmed the presence of intratumoral microbiota in various non-gastrointestinal cancers, including ovarian cancer (Sheng et al., 2023; Yang et al., 2023). However, the functional roles of these intratumoral microbes in ovarian cancer pathogenesis, progression, and clinical outcomes remain poorly understood. Notably, one clinical study reported an association between concurrent antibiotic (ABX) administration and poorer prognosis in ovarian cancer patients (Chambers et al., 2020); yet, the underlying biological mechanisms remain uncharacterized. To date, no study has investigated the potential involvement of intratumoral microbiota in mediating platinum resistance in ovarian cancer. To address this knowledge gap, the present study is the first to comprehensively compare the intratumoral microbial composition between platinum-sensitive and platinum-resistant ovarian cancer patients and to explore plausible mechanistic links between microbial dysbiosis and treatment resistance.

Firstly, the results of our study revealed that some intratumoral microbiota are the specifical microbiome in different groups. Previous studies have found that there were differences between the intratumoral microbiota of ovarian cancer and normal ovarian tissue (Wang et al., 2020). Nevertheless, we firstly demonstrated that the composition of intratumoral microbiota were obviously different among patients with different platinum-sensitive status. At the same time, we found that p_Firmicutes, p_Actinobacteria and p_Acidobacteria were significantly higher in the PR group than in PSR and PRR groups. Genus Lactococcus, Bifidobacterium and Corynebacterium were significantly enriched in PR group compared with PSR and PRR groups. More importantly, Lactococcus lactis at the species level was confirmed to be strongly associated with platinum-refractory status in HGSOC.

One previous study revealed that the phylum Actinobacteria and class Actinobacteria of gut microbiota are risk factors for breast cancer and lung cancer but are protective factors for oral cavity cancer (Long et al., 2023). Dohlman AB, et al. showed that there were a relatively high proportion of Actinobacteria in breast cancer tissue samples (Dohlman et al., 2021). Furthermore, the distribution of Bifidobacterium were significantly different based on clinical stages of cancer, which also revealed the microbiome may be the key factors influence the progression of breast cancer (Bernardo et al., 2023; Taki et al., 2025). As respects of colorectal cancer and advanced pancreatic cancer, there were also higher abundance of Bifidobacterium in the tumor tissues (Nugent et al., 2014). This further suggests that Actinomycetes may play an important role in the tumor occurrence and progression. However, to prove the effect of Actinomycetes in the evolution of the platinum resistance phenotype of ovarian cancer, we are prepared to carry out further research.

Based on platinum sensitivity, comparative analyses across multiple taxonomic levels—including phylum, class, order, family, genus, and species—revealed that the Firmicutes phylum exhibited the highest relative abundance in the platinum-refractory group, followed by the platinum-resistant group, and the lowest abundance in the platinum-sensitive group; these differences were statistically significant. Subsequent sunburst plots and species-level tracking indicated that this signal was predominantly attributable to “Lactococcus”. Linear discriminant analysis effect size (LEfSe) of intratumoral microbiota further demonstrated that “Lactococcus” was specifically enriched in platinum-resistant and platinum-refractory ovarian cancer, with significantly higher abundance compared to the platinum-sensitive group.

“Lactococcus”, a Gram-positive bacterium, employs a homolactic fermentation metabolism, converting carbohydrates into lactic acid (Kleerebezem et al., 2020). Notably, it produces L-lactic acid—a chiral isomer of lactic acid and the predominant endogenous form naturally synthesized and metabolized in humans. Under physiological conditions (pH ≈ 7.4), L-lactic acid exists primarily in its dissociated form (i.e., L-lactate^-^). Due to its negative charge, L-lactate^-^ cannot passively diffuse across lipid bilayers and instead relies on monocarboxylate transporter 1 (MCT1) for transmembrane transport (Jiang et al., 2025).

Accumulating evidence indicates that lactate in the tumor microenvironment (TME) can be shuttled into tumor cells via monocarboxylate transporters (MCTs)—a process termed the “lactate shuttle”—thereby reprogramming tumor cell metabolism and epigenetic landscapes and ultimately promoting chemotherapy resistance and immunotherapy evasion (Chen et al., 2025; He et al., 2025; Kim et al., 2025). Consistent with this, Colbert et al. reported that intratumoral colonization by “Lactobacillus iners”—a relatively inert vaginal commensal—in cervical cancer drives resistance to both chemotherapy and radiotherapy through its metabolic output of L-lactic acid (Colbert et al., 2023). Collectively, these findings suggest that intratumoral “Lactococcus” in ovarian cancer may contribute to platinum resistance by secreting L-lactic acid, thereby remodeling the TME and aberrantly activating lactate-associated signaling pathways in tumor cells. Thus, intratumoral “Lactococcus” represents a promising microbial target for improving therapeutic efficacy in ovarian cancer. Recent studies have revealed that L-lactate—accumulated in tumor cells via the Warburg effect—can induce lactylation modifications on key proteins involved in homologous recombination (HR) DNA repair, thereby enhancing DNA damage repair capacity and driving chemoresistance in tumors.

Additionally, the differences in bacterial metabolism and functional prediction in different platinum-sensitive status were analyzed based on metagenomic PICRUST2. We found that there are also differences in intratumoral microbial function and metabolism in ovarian cancer patients with different platinum sensitivity status. This further demonstrated that intratumoral microorganisms may play an important role in the platinum resistance of ovarian cancer through metabolic differences. Our study revealed that metabolic enzyme NQO1 was highly expressed in PR and PRR groups, which further revealed that intratumoral microbiota may participate in the progression of drug resistance in HGSOC through high expression of NQO1.

Finally, there were several limitations deserving further analysis and discussion. A major limitation was that our research preliminarily explored the relationship between intratumoral microbes and the platinum-sensitive status of ovarian cancer, while the causal relationship and specific mechanism require more rigorous experiments for validation. Another limitation was that we generated the results by using high-throughput sequencing which obtains microbiome information from RNA sequencing data. Additionally, the number of patients included in the study was only 22, and a larger sample of patient specimens is needed for further research. Furthermore, this study preliminarily analyzed the intratumoral microbiota differences in ovarian cancer with different platinum-sensitive status. However, no specific bacteria that play an important role in the evolution of platinum resistance have been fully confirmed. Further exploration is needed to confirm the microorganisms that play an important role in tumor cells. This study has some limitations but opens new avenues to explore the intratumoral microbiome-metabolome associations for biomarker discovery.

## Conclusions

This study is pioneering in identifying variations in intratumoral microbiota associated with differing sensitivities to platinum therapy in ovarian cancer patients. Intratumoral microbiota-based biomarkers may be helpful in exploring the novel mechanism of platinum resistance in ovarian cancer and providing new avenue for treating this disease.

## Data Availability

The datasets presented in this study can be found in online repositories. The names of the repository/repositories and accession number(s) can be found below: https://ngdc.cncb.ac.cn/omix, PRJCA033363, no.OMIX008191.

## Supplementary material

Supplementary Figure 1 Relative bacterial evenness was evaluated by rank abundance curves, exhibiting similar patterns in all samples.

Supplementary Figure 2 Analysis at the class, order, family, genus, and species levels was also performed.

Supplementary Figure 3 Bacterial abundance at the genus level was also compared between three groups.

Supplementary Figure 4 Based on LefSe analysis, we also found that PR ovarian cancer had a richer intratumoral microbiota, followed by the PRR group PSR group.

Supplementary Figure 5 PICRUST analysis identified 30 EC pathways **(Figure S5)**, 30 KO pathways **(Figure S6)** and 30 TIGRFAM pathways **(Figure S7)** with significant differential abundance between these three cohorts.

Supplementary Figure 6 PICRUST analysis identified 30 EC pathways **(Figure S5)**, 30 KO pathways **(Figure S6)** and 30 TIGRFAM pathways **(Figure S7)** with significant differential abundance between these three cohorts.

Supplementary Figure 7 PICRUST analysis identified 30 EC pathways **(Figure S5)**, 30 KO pathways **(Figure S6)** and 30 TIGRFAM pathways **(Figure S7)** with significant differential abundance between these three cohorts.

Supplementary Figure 8 Phenotypic analysis based on BugBase were also conducted, the phenotypes of which are divided into nine types: aerobic, anaerobic, mobile element-containing, facultative anaerobic, biofilm formation, Gram-negative, Gram-positive, potential pathogenic and stress tolerance **(A, B)**.
