# Supplementary figures and images for: Investigating the diversity of intratumoral microbiota in high-grade serous ovarian cancer with varying platinum sensitivity
Source: Front Cell Infect Microbiol. 2026 Jun 4;16:1652322. doi: 10.3389/fcimb.2026.1652322 (PMC13275416; doi:10.3389/fcimb.2026.1652322)

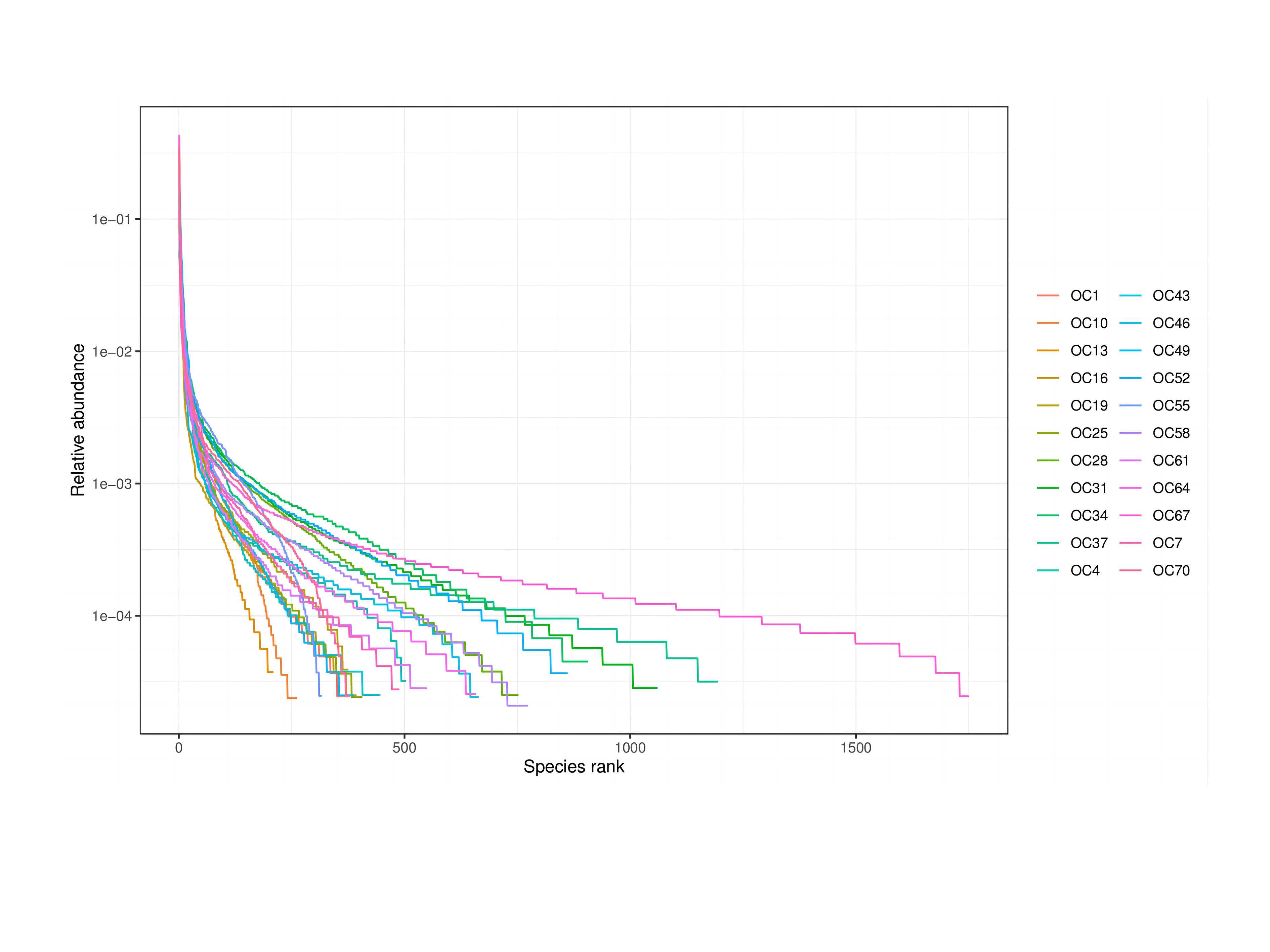

Supplement: Supplementary Figure 1 — Relative bacterial evenness was evaluated by rank abundance curves, exhibiting similar patterns in all samples. [file Image1.jpeg]

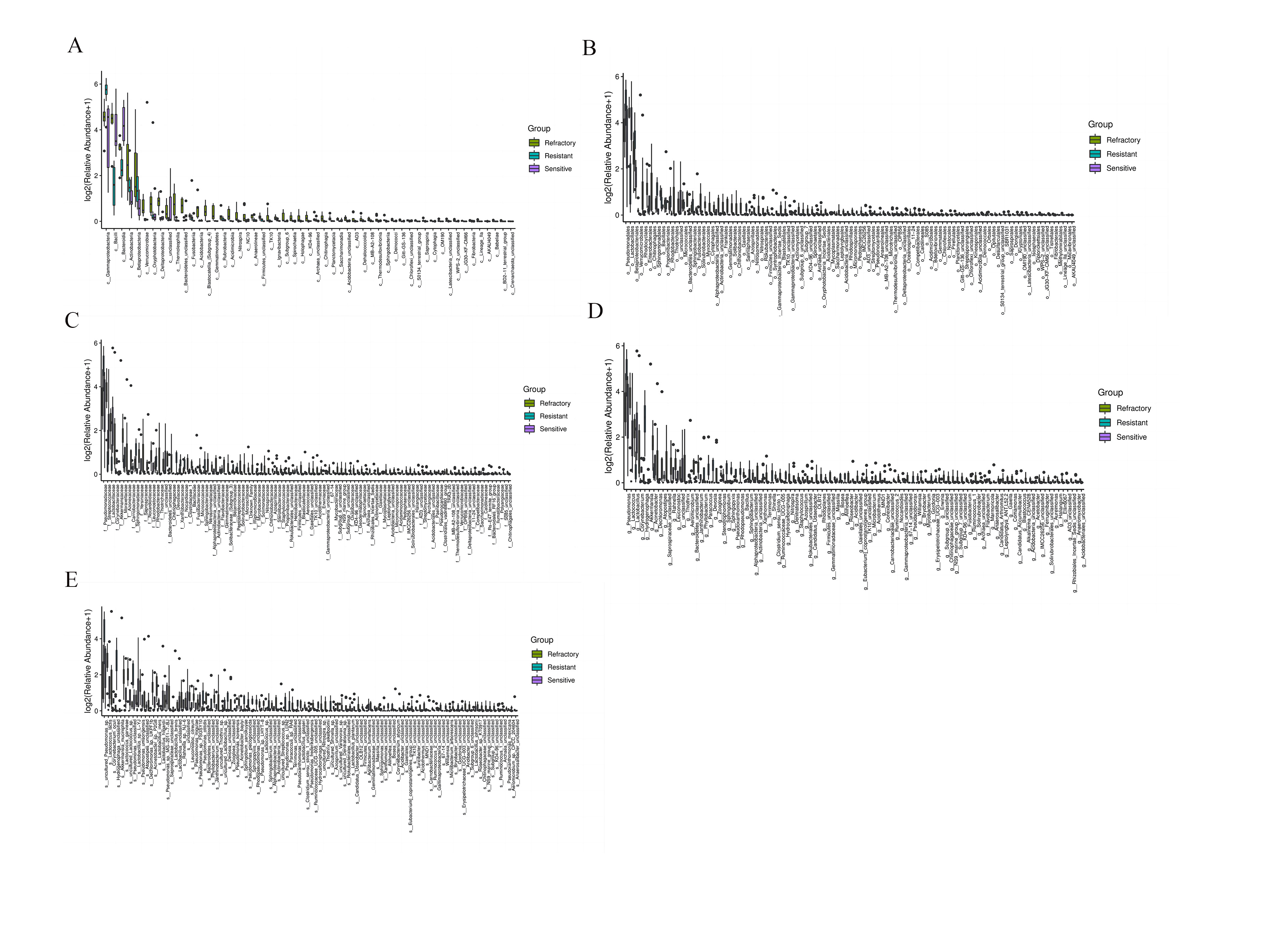

Supplement: Supplementary Figure 2 — Analysis at the class, order, family, genus, and species levels was also performed. [file Image2.jpeg]

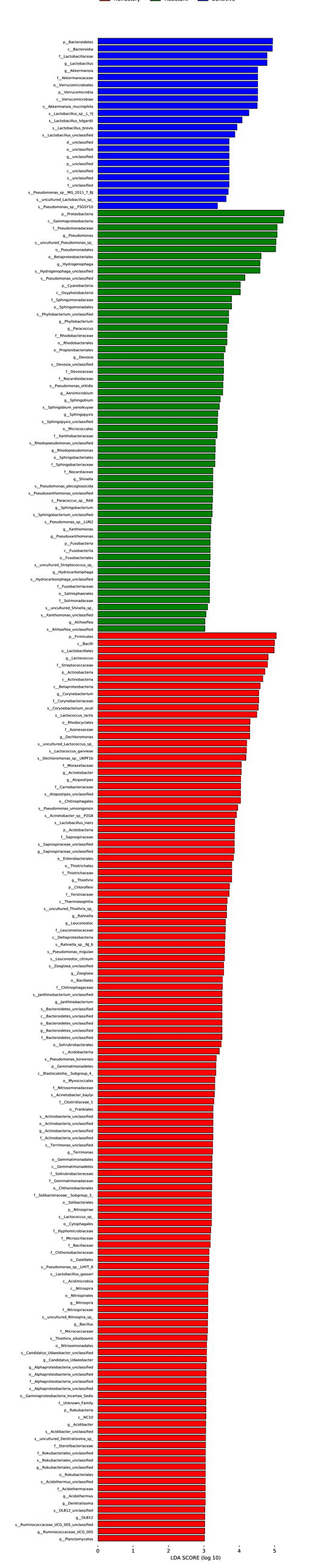

Supplement: Supplementary Figure 3 — Bacterial abundance at the genus level was also compared between three groups. [file Image3.jpeg]

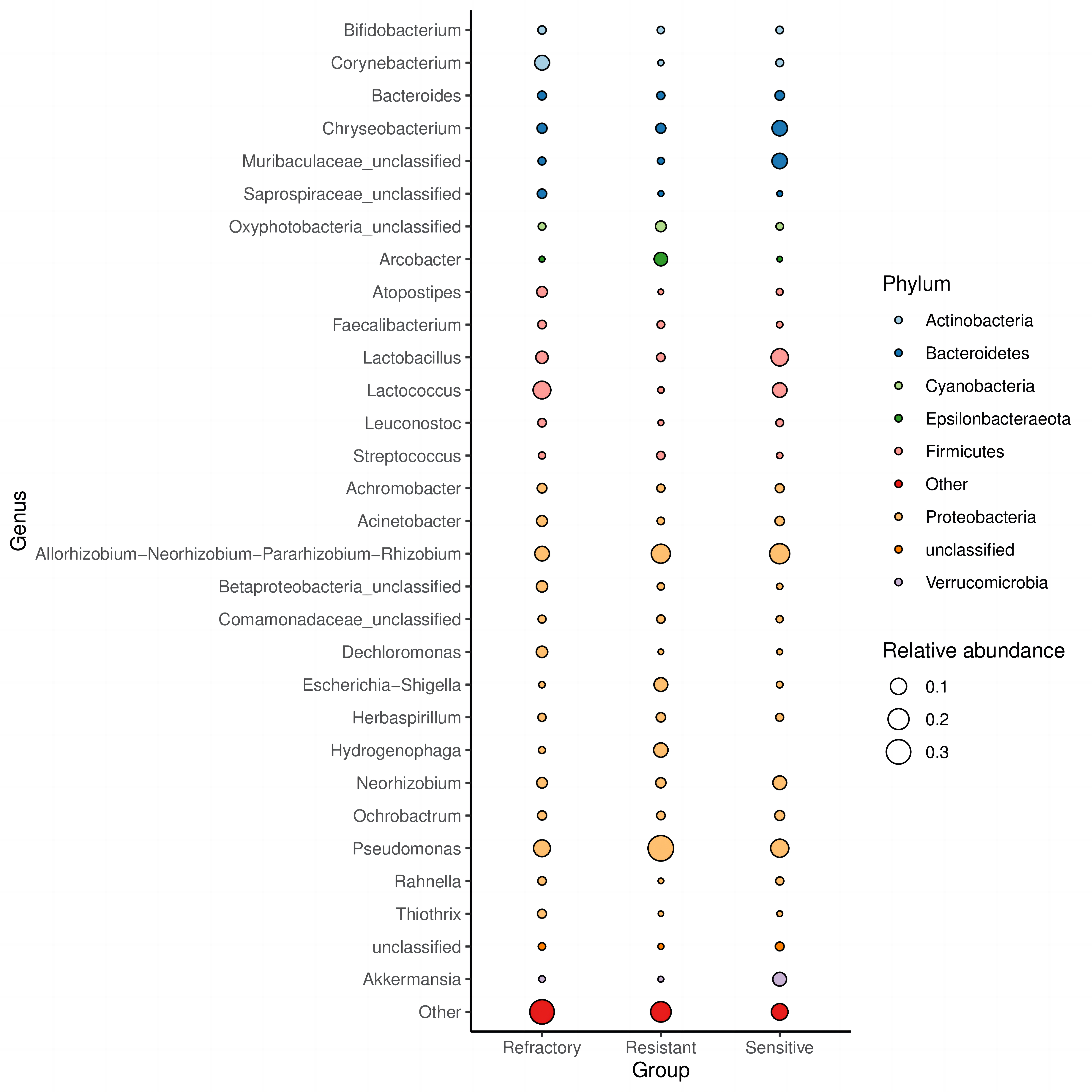

Supplement: Supplementary Figure 4 — Based on LefSe analysis, we also found that PR ovarian cancer had a richer intratumoral microbiota, followed by the PRR group PSR group. [file Image4.jpeg]

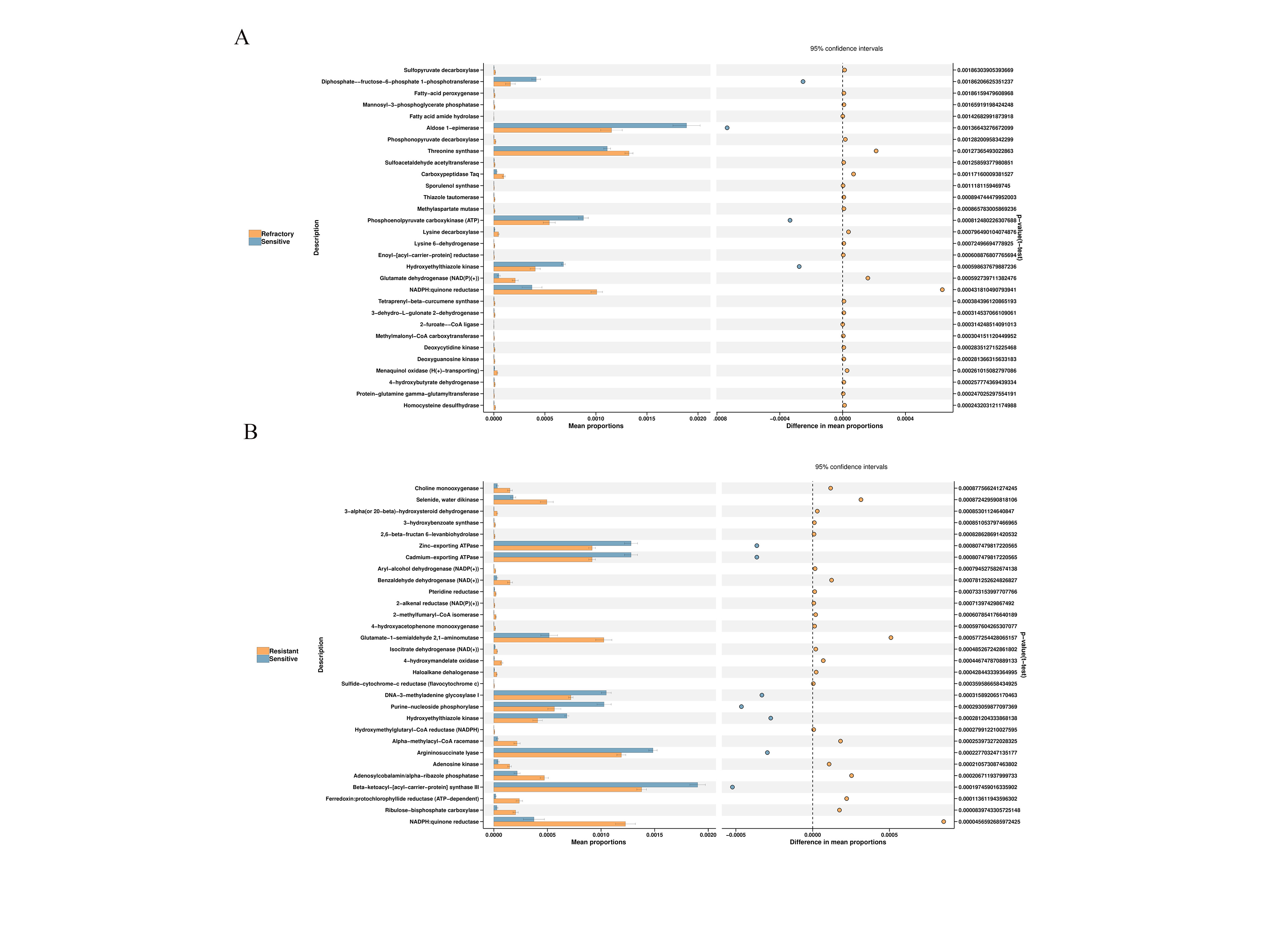

Supplement: Supplementary Figure 5 — PICRUST analysis identified 30 EC pathways (Figure S5), 30 KO pathways (Figure S6) and 30 TIGRFAM pathways (Figure S7) with significant differential abundance between these three cohorts. [file Image5.jpeg]

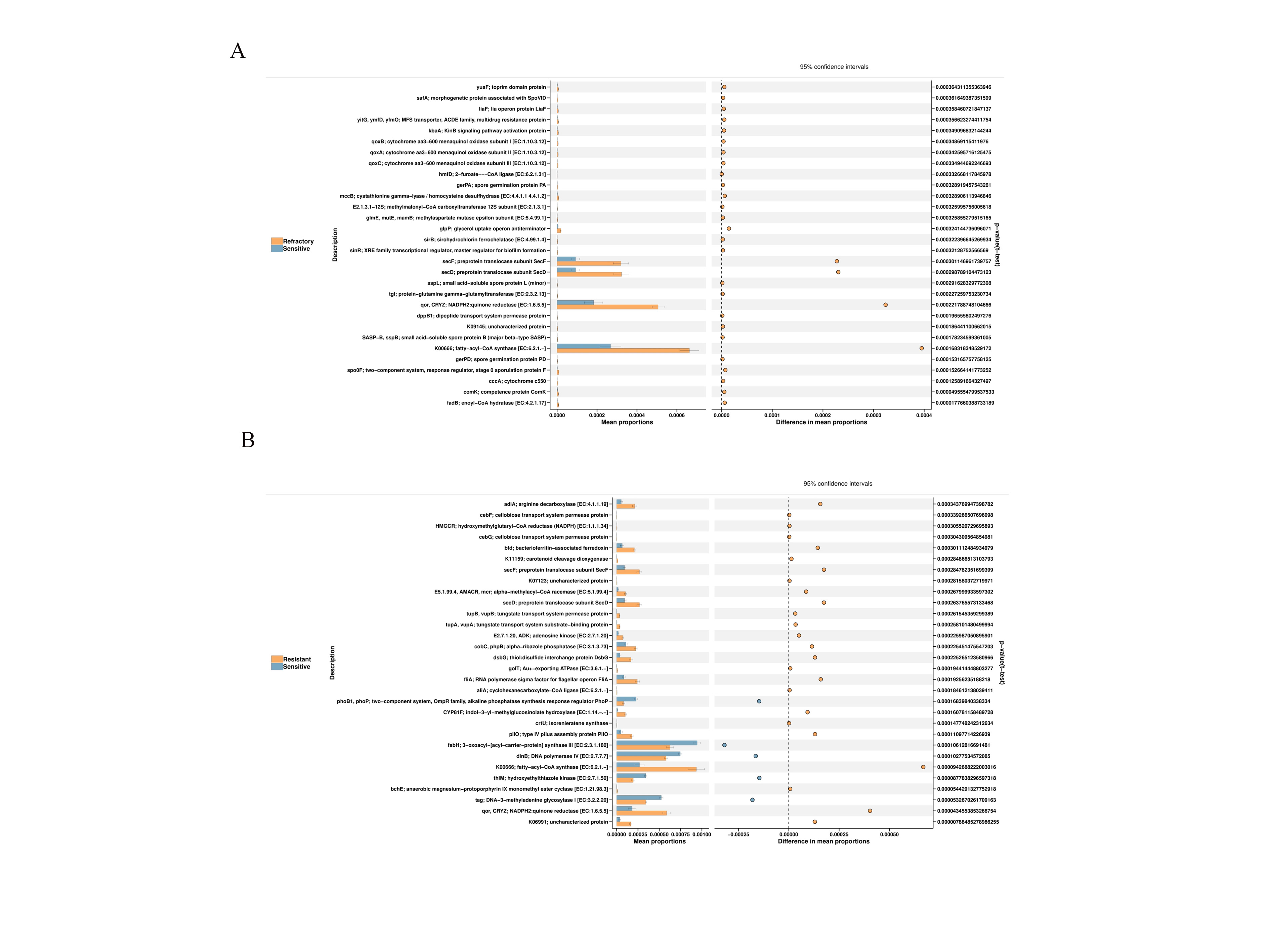

Supplement: Supplementary Figure 6 — PICRUST analysis identified 30 EC pathways (Figure S5), 30 KO pathways (Figure S6) and 30 TIGRFAM pathways (Figure S7) with significant differential abundance between these three cohorts. [file Image6.jpeg]

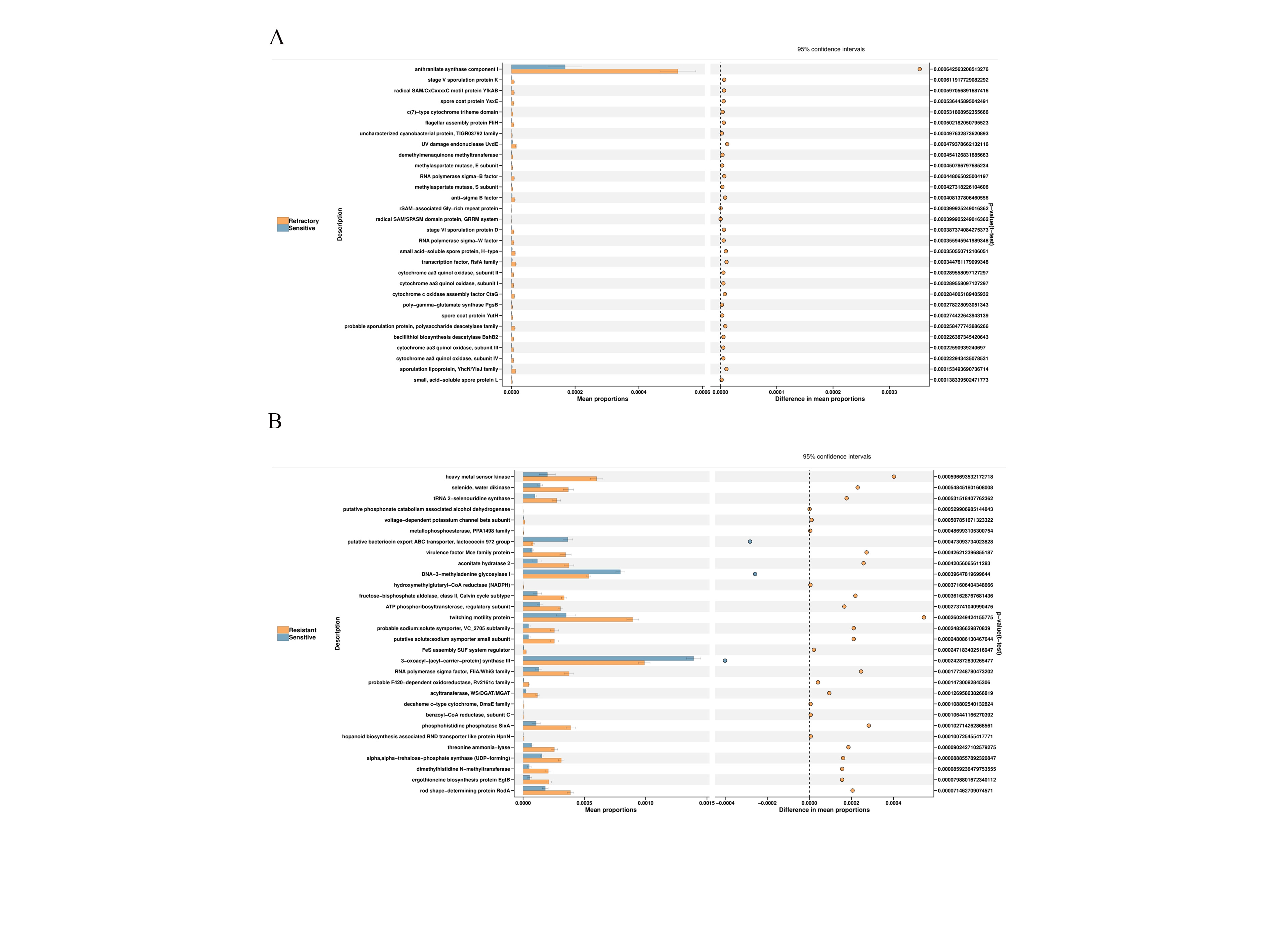

Supplement: Supplementary Figure 7 — PICRUST analysis identified 30 EC pathways (Figure S5), 30 KO pathways (Figure S6) and 30 TIGRFAM pathways (Figure S7) with significant differential abundance between these three cohorts. [file Image7.jpeg]

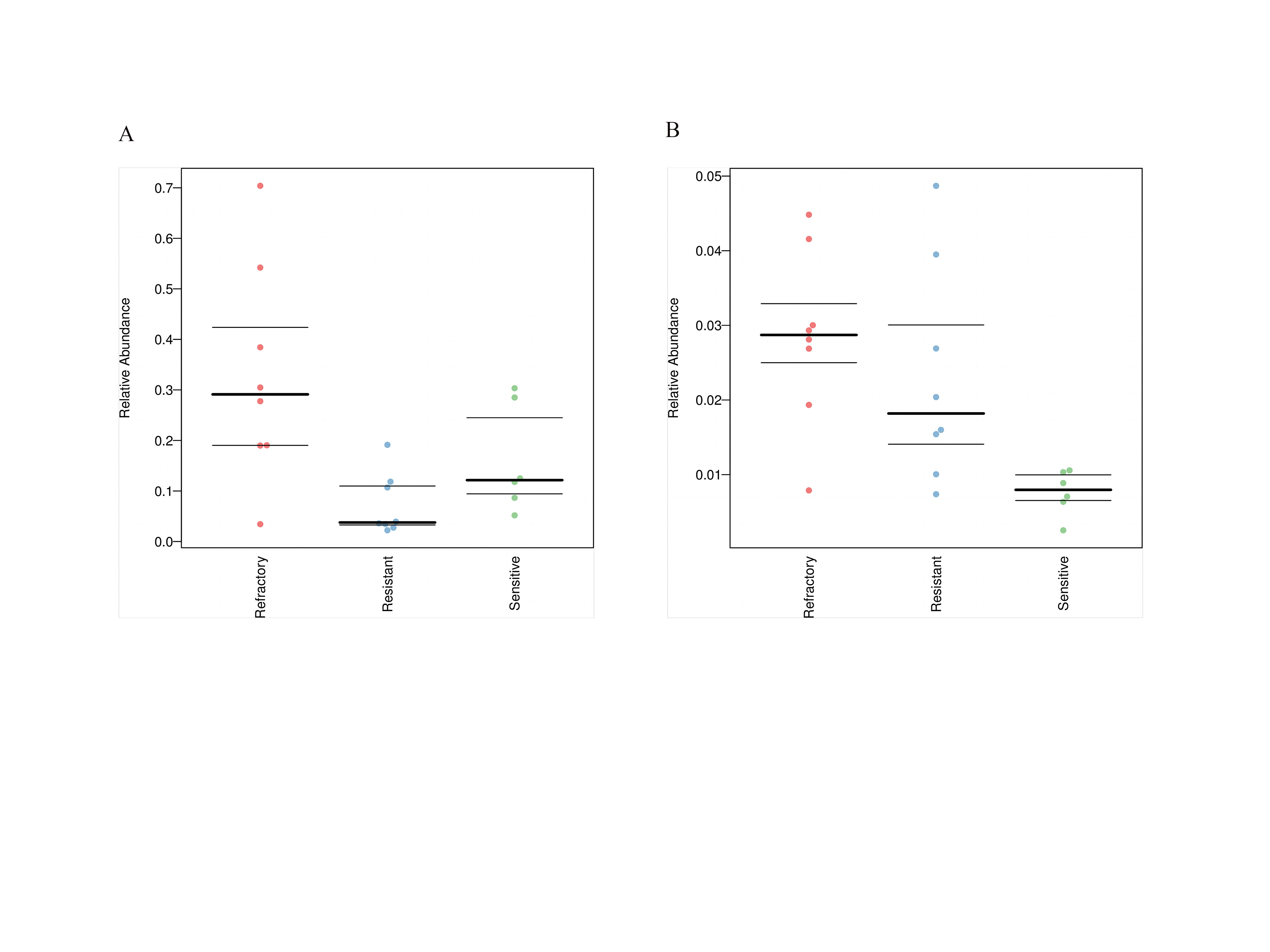

Supplement: Supplementary Figure 8 — Phenotypic analysis based on BugBase were also conducted, the phenotypes of which are divided into nine types: aerobic, anaerobic, mobile element-containing, facultative anaerobic, biofilm formation, Gram-negative, Gram-positive, potential pathogenic and stress tolerance (A, B). [file Image8.jpeg]
